# Recent advances in the use of PI3K inhibitors for glioblastoma multiforme: current preclinical and clinical development

**DOI:** 10.1186/s12943-017-0670-3

**Published:** 2017-06-07

**Authors:** Hua-fu Zhao, Jing Wang, Wei Shao, Chang-peng Wu, Zhong-ping Chen, Shing-shun Tony To, Wei-ping Li

**Affiliations:** 1 Department of Neurosurgery & Shenzhen Key Laboratory of Neurosurgery, the First Affiliated Hospital of Shenzhen University, Shenzhen Second People’s Hospital, Shenzhen, 518035 China; 2 Department of Neurosurgery/Neuro-oncology, Sun Yat-sen University Cancer Center, State Key Laboratory of Oncology in South China, Collaborative Innovation Center for Cancer Medicine, Guangzhou, 510060 China; 3 Department of Health Technology and Informatics, The Hong Kong Polytechnic University, Hong Kong, China; 4 College of Clinical Medicine, Anhui Medical University, Hefei, 230032 China

**Keywords:** Glioblastoma, GBM, PI3K, mTOR

## Abstract

Glioblastoma multiforme (GBM) is the most common and aggressive malignant primary tumor in the central nervous system. One of the most widely used chemotherapeutic drugs for GBM is temozolomide, which is a DNA-alkylating agent and its efficacy is dependent on *MGMT* methylation status. Little progress in improving the prognosis of GBM patients has been made in the past ten years, urging the development of more effective molecular targeted therapies. Hyper-activation of the phosphatidylinositol 3-kinase (PI3K)/Akt pathway is frequently found in a variety of cancers including GBM, and it plays a central role in the regulation of tumor cell survival, growth, motility, angiogenesis and metabolism. Numerous PI3K inhibitors including pan-PI3K, isoform-selective and dual PI3K/mammalian target of rapamycin (mTOR) inhibitors have exhibited favorable preclinical results and entered clinical trials in a range of hematologic malignancies and solid tumors. Furthermore, combination of inhibitors targeting PI3K and other related pathways may exert synergism on suppressing tumor growth and improving patients’ prognosis. Currently, only a handful of PI3K inhibitors are in phase I/II clinical trials for GBM treatment. In this review, we focus on the importance of PI3K/Akt pathway in GBM, and summarize the current development of PI3K inhibitors alone or in combination with other inhibitors for GBM treatment from preclinical to clinical studies.

## Background

Glioblastoma multiforme (GBM), classified as WHO grade IV glioma, is the most common, aggressive and malignant primary tumor in the central nervous system [1]. The incidence rate of GBM is the highest in malignant brain tumors, with 3.19 new cases per 100,000 populations per year [1, 2]. Glioblastoma is characterized by rapid growth, extensive infiltration to neighboring brain tissues, pseudopalisading necrosis and angiogenesis, which contribute to poor prognosis. Despite the standard treatment including maximal surgical resection followed by adjuvant radiotherapy and chemotherapy, the prognosis of GBM patients remains poor. The 2-year relative survival rate for GBM patients is approximately 26.5%, and the median overall survival is 14.6 months [3].

**Fig. 1:**
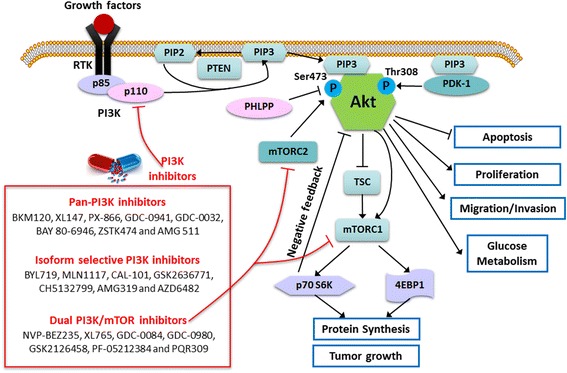
Schematic diagram of PI3K/Akt/mTOR signaling pathway and relevant PI3K inhibitors. When the growth factors bind to their corresponding RTKs, the regulatory isoform of PI3K (p85) binds to RTKs and relieves its inhibition on the catalytic isoform (p110), leading to the activation of PI3K. PI3K gives rise to the production of the lipid messenger PIP3 from PIP2, which can be reversed by the tumor suppressor PTEN. Subsequently, PIP3 binds to the PH domain of Akt and recruits Akt to the plasma membrane. PDK-1 is also recruited by PIP3 to the plasma membrane through its PH domain, and then phosphorylates Akt at Thr308. Akt is completely activated through phosphorylation at Ser473 by mTORC2 (PDK-2). PHLPP is able to dephosphorylate Akt at Ser473. Activated Akt phosphorylates a variety of downstream pathway molecules to facilitate tumor cell survival, proliferation, migration, invasion and glucose metabolism. The Akt downstream mTORC1 axis also regulates protein synthesis and cell growth. The tumor suppressor TSC1 and TSC2 are two substrates of Akt, and their phosphorylation leads to the blockade of their inhibitory effects on mTORC1. Activated mTORC1 then phosphorylates 4EBP1 and p70 S6 K, resulting in the initiation of protein translation. Activation of mTORC1 also triggers a negative feedback to suppress Akt phosphorylation. Numerous PI3K inhibitors have been developed to target PI3K/Akt/mTOR signaling. Pan-PI3K and isoform-selective PI3K inhibitors suppress the activities of p110 catalytic isoforms, while PI3K/mTOR inhibitors block the activation of both p110 and mTORC1/2

Genetic aberrations in glioblastoma including *EGFR*, *PDGFRA, PIK3CA*, *PTEN, TP53* and *CDKN2A/B* etc., drive the dysfunction of signaling pathways such as PI3K/Akt/mTOR, p53 and RB1 pathways, and open up possible therapies for GBM by targeting these pathways with selective inhibitors [4]. The phosphatidylinositol 3-kinases (PI3Ks)/Akt signaling pathway plays a central role in the regulation of signal transduction, which mediates various biological processes including cell proliferation, apoptosis, metabolism, motility and angiogenesis in GBM. Generally, activation of PI3K/Akt pathway starts with activation of receptor tyrosine kinases (RTKs) or G protein-coupled receptors (GPCRs). Class I_A_ and I_B_ PI3Ks mainly respond to the activation of RTKs and GPCRs, respectively. Epidermal growth factor receptor (EGFR, ErbB-1), a transmembrane protein, belongs to a RTK subfamily – ErbB family. After binding to its ligand EGF, EGFR undergoes a transition from an inactive monomeric form to an active homodimer. Its variant III mutation (EGFRvIII), characterized by an in-frame deletion in exons 2–7, is common (25%–50%) in GBM and produces a truncated EGFR protein without the extracellular ligand-binding domain, leading to its ligand-independent constitutive activation [5]. A simplified schematic diagram showing PI3K/Akt signaling is presented in Fig. 1. When a ligand such as EGF or PDGF binds to its corresponding RTK, the intracellular C-terminal kinase domain of RTK undergoes conformational alterations and autophosphorylation, which provides binding sites for the regulatory subunits of PI3K. The interaction between RTK and PI3K regulatory subunits subsequently relieves the inhibitory effect on the catalytic subunits, leading to elevated lipid kinase activity of PI3K. Activation of PI3K transforms phosphatidylinositol 4,5-bisphosphate (PtdIns(4,5)P2, PIP2) to phosphatidylinositol 3,4,5-triphosphate (PtdIns(3,4,5)P3, PIP3) in plasma membrane. Subsequently, PIP3 binds to Akt and anchors it to the plasma membrane. Akt at Thr308 and Ser473 residues are then phosphorylated by phosphoinositide-dependent kinase −1 (PDK-1) and mammalian target of rapamycin complex 2 (mTORC2), respectively, leading to its complete activation. PTEN (phosphatase and tensin homolog deleted on chromosome 10) and PHLPP (PH domain and leucine rich repeat protein phosphatase) are two tumor suppressors, the former transforms PIP3 to PIP2 and blocks the recruitment of Akt to the plasma membrane, while the latter dephosphorylates Ser473 in Akt and subsequently suppresses Akt activation [6, 7]. Activated Akt, in turn, phosphorylates downstream pathway molecules to mediate metabolism, cell growth, angiogenesis, motility and apoptosis [8]. It mediates protein synthesis by phosphorylating tuberous sclerosis complex (TSC) and then activating mTOR. mTOR and its partner Raptor (mTORC1) bind to p70 S6 K and eukaryotic initiation factor 4E–binding protein 1 (4EBP1), leading to their phosphorylation and initiation of protein translation [9].

Hyper-activation of PI3K/Akt pathway confers rapid growth, tumor progression and multidrug resistance upon GBM cells. Inhibition of PI3K alone or in combination with other molecules may result in GBM cell death and retarded tumor progression. Here, we summarize the roles of PI3K in GBM and highlight recent advances and challenges in the development of PI3K inhibitors as targeted therapeutic agents for GBM. The most recent experimental, preclinical and clinical studies are presented to emphasize the prospect of PI3K inhibitors for GBM treatment.

### Roles of PI3K catalytic isoforms in glioblastoma

Class I_A_ PI3K is composed of a heterodimer consisting of a 110 kDa catalytic subunit (p110α, p110β and p110δ) and an 85 kDa regulatory subunit [8]. The regulatory p84/p101 and catalytic p110γ subunits form the only member in the class I_B_ PI3K. The p110α and p110β isoforms are ubiquitously expressed, whereas p110δ and p110γ are primarily expressed in leukocytes. The p110α, p110β and p110δ isoforms exhibit distinct roles in different pathological processes in cancer cells. The p110α isoform is required for tumor cell proliferation, migration and invasion, whereas p110β is essential to cell survival and tumorigenesis [10–12].

Compared to *PTEN* or *EGFR* mutations, mutations of *PIK3CA* (encodes p110α) in 10 of 20 exons are less frequent but very important in GBM, ranging from 4% to 27% [13–16]. Whole genome sequencing of TCGA GBM samples shows that 18.3% of GBM exhibit *PIK3CA* and *PIK3R1* mutations, which are mutually exclusive of *PTEN* mutation or deletion [4]. The majority of *PIK3CA* mutants (including the hot-spot mutations in exon 9 and 20) show gain of PI3K function and promote the recruitment of p110α to membrane phospholipids, leading to the constitutive activation of p110α and Akt [17]. Therefore, p110α may be a promising target for GBM treatment when harboring *PIK3CA* mutation. Accumulating studies demonstrate that p110α plays a critical role in tumorigenesis and progress of GBM. Knockdown of *PIK3CA* significantly inhibits cell viability, migration and invasion in GBM cells through decreasing Akt and FAK activation [11]. In addition, GBM cell growth, survival and migration are effectively suppressed in vitro by the p110α isoform-selective inhibitors A66 or PIK-75 [18–20].

Although somatic mutations of the p110β isoform have rarely been identified in GBM and other cancers, clinical studies show that overexpression of p110β is found in 15% of invasive breast cancer (48 of 315 cases) and 28% of colorectal cancer (23 of 82 cases), and is correlated with poor overall survival, with one study showing that p110β overexpression is barely detected in GBM samples (1 of 74 cases) [20–22]. In contrast to the patient samples, p110β overexpression is frequently found in a series of GBM cell lines [20, 23]. Nevertheless, p110β plays a crucial role in GBM cell growth, survival and migration in the context of *PTEN* loss. Knockdown of *PIK3CB* (encodes p110β) suppresses cell proliferation and induces caspase-dependent apoptosis in GBM in vitro and in vivo, and it synergizes with PTEN restoration [23, 24]. However, *PIK3CB* knockdown barely suppresses GBM cell migration [23]. On the contrary, inhibition of p110β using the selective inhibitor TGX-221 significantly suppresses cell migration in GBM cells, with little inhibitory effect on GBM cell survival and invasion [19, 20]. These findings suggest a kinase-independent function of p110β and a competition model that p110β modulates PI3K activity by competing against p110α for RTK binding in GBM [25]. Moreover, PTEN-deficient tumor growth depends on p110β rather than p110α, suggesting that targeting p110β may be an option for the treatment of PTEN-deficient GBM [26].

Due to the high expression level in leukocytes, the p110δ and p110γ subunits are promising therapeutic targets for hematologic malignancies including leukemia, lymphoma and multiple myeloma [27, 28]. Recently, overexpression of p110δ is also observed in solid tumors such as GBM, neuroblastoma, breast and prostate cancers, indicating that inhibition of p110δ may also be an attractive option for GBM treatment [23, 29, 30]. Our previous study shows that p110δ is overexpressed in 50% of high grade glioma cell lines (6 of 12), and knockdown of p110δ inhibits migration and invasion of GBM cells by decreasing focal adhesion kinase (FAK) expression [23]. However, inhibition of p110δ by its selective inhibitor CAL-101 moderately impairs GBM cell proliferation and migration, but it doesn’t significantly suppress tumor growth in GBM xenograft mouse model [19]. These findings indicate that targeting p110δ may not be an effective approach for GBM treatment. On the other hand, accumulating evidence indicates that p110γ is also involved in the development and progression of solid tumors. CXCR4-induced activation of p110γ leads to increased migration, invasion and metastasis of melanoma and breast cancer cells [31, 32]. A sensitized RNA interference screen identifies that p110γ is vital to medulloblastoma cell proliferation and confer resistance of these cells to cisplatin [33]. Unfortunately, to date there is no study describing the role of p110γ in GBM.

### PI3K inhibitors and their therapeutic potentials for GBM

According to their isoform selectivity, PI3K inhibitors are generally classified into pan-PI3K, isoform-selective and dual PI3K/mTOR inhibitors. Currently, more than 50 PI3K inhibitors have been designed and produced for cancer treatment. Characteristics and structural formulas of the most commonly used and novel PI3K inhibitors are listed in Tables 1 and 2. However, only a handful of them such as BKM120, XL147 and XL765 have successfully entered into clinical trials for GBM treatment. The prevalent PI3K inhibitors with great potential in clinical practice for GBM are discussed below.

**Table 1:** Characteristics of novel PI3K inhibitors

Classification	Drug name	IC_50_ (nM)					IC_50_ to GBM cells (μM)	References
		p110α	p110β	p110δ	p110γ	mTORC1/2		
Pan-PI3K inhibitors	Pictilisib (GDC-0941)	3	33	3	75	580	0.95 (U-87 MG)	[63, 130]
	Taselisib (GDC-0032)	0.29	9.1	0.12	0.97	1200	N/A	[131]
	Buparlisib (BKM120)	52	166	116	262	4600	1.28 ± 0.33 (p53 wt cells) 2.08 ± 0.69 (p53 mutant/deleted cells)	[35, 36]
	Pilaralisib (XL147, SAR245408)	39	383	36	23	>15,000	24.0 (U-87 MG) >30.0 (U-251 and U-373 MG)	[43]
	Copanlisib (BAY 80–6946)	0.5	3.7	0.7	6.4	45	~0.1 (U-87 MG)	[73, 132]
	Sonolisib (PX-866)	0.1	>300	2.9	N/A	N/A	N/A	[52]
	ZSTK474	16	44	4.6	49	>10,000	7.0 (U-87 MG) 1.1 (U-251 MG)	[68, 133]
	AMG 511	4	6	2	1	N/A	N/A	[70]
Isoform-selective inhibitors	Alpelisib (BYL719)	5	1200	290	250	>9100	N/A	[75]
	Idelalisib (CAL-101, GS-1101)	820	565	2.5	89	>1000	39.11 (U-87 MG)	[19, 134]
	AMG319	3300	2700	18	850	N/A	N/A	[135]
	AZD6482	870	10	80	1090	N/A	N/A	[136]
	CH5132799	14	120	500	36	1600	N/A	[137]
	AS-605240	60	270	300	8	N/A	N/A	[138]
	MLN1117 (INK1117)	15	4500	1900	13,390	1670	N/A	[139]
Dual PI3K/mTOR inhibitors	Dactolisib (NVP-BEZ235)	4	75	7	5	6	0.04–0.1	[89, 140]
	NVP-BGT226	4	63	N/A	38	N/A	N/A	[141]
	Omipalisib (GSK2126458, GSK458)	0.019	0.13	0.024	0.06	0.18/0.3	N/A	[142]
	GSK1059615	0.4	0.6	2	5	12	N/A	[143]
	Voxtalisib (XL765, SAR245409)	39	113	41	9	160/910	2.6 (U-87 MG) 19.6 (U-251 MG) >30 (U-373 MG)	[100]
	Apitolisib (GDC-0980)	5	27	7	14	17	N/A	[144]
	GDC-0084 (RG7666)	2	46	3	10	70	0.74 (U-87 MG)	[104]
	VS-5584 (SB2343)	16	68	42	25	37	0.18 (U-87 MG)	[145]
	PF-04691502	1.8	2.1	1.6	1.9	16	N/A	[146]
	Gedatolisib (PF-05212384, PKI-587)	0.4	N/A	N/A	5.4	1.6	N/A	[147]
	PKI-402	2	7	14	16	3	0.077 (U-87 MG)	[148]

N/A, not available

**Table 2:** Structural formulas of PI3K inhibitors

Classification	Compound	Structural formula	Compound	Structural formula
Pan-PI3K inhibitors	Pictilisib (GDC-0941)		Copanlisib (BAY 80–6946)	
	Taselisib (GDC-0032)		Sonolisib (PX-866)	
	Buparlisib (BKM120)		ZSTK474	
	Pilaralisib (XL147, SAR245408)		AMG 511	
Isoform-selective inhibitors	Alpelisib (BYL719)		CH5132799	
	Idelalisib (CAL-101, GS-1101)		AS-605240	
	AMG319		MLN1117 (INK1117)	
	AZD6482			
Dual PI3K/mTOR inhibitors	Dactolisib (NVP-BEZ235)		GDC-0084 (RG7666)	
	NVP-BGT226		VS-5584 (SB2343)	
	Omipalisib (GSK2126458, GSK458)		PF-04691502	
	GSK1059615		Gedatolisib (PF-05212384, PKI-587)	
	Voxtalisib (XL765, SAR245409)		PQR309	
	Apitolisib (GDC-0980)			

### Pan-PI3K inhibitors

The first generation of pan-PI3K inhibitors wortmannin and LY294002, that target all class I_A_ p110 isoforms, are of limited use clinically due to their poor pharmaceutical properties (insolubility and short half-life), off-target effects and unacceptable toxicities in animal studies [34]. A new generation of pan-PI3K inhibitors with improved safety, efficacy and pharmacokinetics such as BKM120, XL147, PX-866, GDC-0941 and GDC-0032 have been developed and have entered into clinical trials (Table 3).

**Table 3:** Current status of PI3K inhibitors in clinical trials (data from http://clinicaltrials.gov)

Classification	Drug name	Tumor(s)	Clinical trials
Pan-PI3K inhibitors	Pictilisib (GDC-0941)	Breast cancer, NSCLC, NHL, GBM	Phase I/II
	Taselisib (GDC-0032)	Breast, ovarian, uterus and squamous cell lung cancers, NSCLC, NHL, lymphoma	Phase I-III
	Buparlisib (BKM120)	Breast, prostate, endometrial, cervical, esophageal, ovarian, colorectal, and H&N cancers, GBM, NSCLC, GIST, RCC, melanoma, lymphoma, leukemia,	Phase I-III
	Pilaralisib (XL147, SAR245408)	Breast, endometrial, and ovarian cancers, GBM, NSCLC, lymphoma	Phase I/II
	Copanlisib (BAY 80–6946)	Endometrial, and H&N cancers, cholangiocarcinoma, lymphoma, NHL	Phase I-III
	Sonolisib (PX-866)	Prostate, colorectal, and H&N cancers, melanoma, NSCLC, GBM	Phase I/II
	ZSTK474	Solid tumors	Phase I
Isoform-selective inhibitors	Alpelisib (BYL719)	Breast, ovarian, gastric, pancreatic, colorectal, H&N, and rectal cancers, melanoma, ESCC, NSCLC, RCC, GIST, MM	Phase I/II
	Idelalisib (CAL-101, GS-1101)	CLL, AML, NHL, MM, and other hematologic malignancies	Phase I-IV
	AMG319	H&N cancer, CLL, lymphoma	Phase I/II
	CH5132799	Solid tumors	Phase I
	MLN1117 (INK1117)	RCC, solid tumors	Phase I/II
Dual PI3K/mTOR inhibitors	Dactolisib (NVP-BEZ235)	Breast, prostate, and endometrial cancer, pancreatic neuroendocrine tumor, RCC, GBM, leukemia	Phase I/II
	NVP-BGT226	Breast cancer, solid tumors	Phase I/II
	Omipalisib (GSK2126458, GSK458)	Solid tumors	Phase I
	GSK1059615	Lymphoma, solid tumors	Phase I
	Voxtalisib (XL765, SAR245409)	Breast, and ovarian cancers, NSCLC, lymphoma, GBM	Phase I/II
	Apitolisib (GDC-0980)	Breast, prostate, and endometrial cancers, RCC, NHL	Phase I/II
	GDC-0084 (RG7666)	High-grade gliomas	Phase I
	VS-5584 (SB2343)	Malignant mesothelioma, lymphoma, advanced non-hematologic malignancies	Phase I
	PF-04691502	Breast, and endometrial cancers	Phase I/II
	Gedatolisib (PF-05212384, PKI-587)	Breast, endometrial, colorectal, ovarian, and H&N cancers, NSCLC, SCLC, AML	Phase I/II
	PQR309	Breast cancer, GBM, lymphoma	Phase I/II

*NHL* Non-Hodgkin’s lymphoma, *CLL* chronic lymphocytic leukemia, *AML* acute myeloid leukemia, *MM* multiple myeloma, *NSCLC* non-small cell lung cancer, *SCLC* small cell lung cancer, *GBM* glioblastoma, *GIST* gastrointestinal stromal tumor, *H&N* head and neck cancer, *RCC* renal cell cancer, *ESCC* esophageal squamous cell carcinoma

### BKM120

Buparlisib (NVP-BKM120, BKM120) is an orally bioavailable pan-PI3K inhibitor against all p110 isoforms with half maximal inhibitory concentration (IC_50_) of 52–262 nM. Its half-life across species in mouse, rat, dog and monkey is 1.6, 11, 6.6 and 3.6 h, respectively [35]. BKM120 induces G_2_/M cell cycle arrest and apoptosis in GBM cells through microtubule misalignment and mitotic dysfunction in a p53-dependent manner [36]. It also promotes tumor necrosis factor-related apoptosis inducing ligand (TRAIL)- and Bcl-2 inhibitor-induced apoptosis in GBM cells via elevated Noxa expression, sequestration of Mcl-1 by Noxa and release of pro-apoptotic protein Bim and Bak from Mcl-1 [37, 38]. Furthermore, preclinical studies show that BKM120 impedes intracerebral U-87 MG GBM cell xenograft growth and prolongs survival of animals harboring xenograft, without obvious adverse effects [36, 39]. Currently, BKM120 is the most frequently-used PI3K inhibitor in the clinical trials for GBM treatment, since it is well-tolerated and permeable to the blood–brain barrier (BBB). In a phase II study of BKM120 in recurrent GBM, patients with radiologic progression and activation of PI3K/Akt pathway including *PIK3CA* mutations, *PTEN* loss and increased phosphorylation of Akt were recruited (NCT01339052). Mild treatment-related toxicities including increased asymptomatic lipase and alanine aminotransferase (ALT)/aspartate transaminase (AST), rash, hyperglycemia and fatigue were seen. Paradoxically, patients harboring *PTEN* loss and/or *PIK3CA* mutations were not sensitive to BKM120 treatment even though it inhibits Akt phosphorylation [40]. Phase I and II multicenter studies on the combination of BKM120 and bevacizumab in patients with relapsed/refractory GBM are ongoing. Generally, this combination treatment was well-tolerated with mild toxicities such as fatigue, hyperglycemia and ALT elevation. Appropriately 26.5% of patients (18 of 68) had stable disease. Median progression-free survival (PFS) and overall survival (OS) were 5.3 and 10.8 months, respectively. Overall response ratio (RR) was 32.3% (22 of 68), including 2 complete remission (CR) and 20 partial remission (PR) (NCT01349660) [41, 42]. There are 4 ongoing or completed clinical trials of BKM120 combined with other drugs in patients with newly diagnosed or recurrent GBM. In a phase Ib dose escalation study, BKM120 was combined with LDE225, a Smoothened (Smo) antagonist, in the patients with advanced solid tumors including recurrent GBM (NCT01576666). Combination of BKM120 and Capmatinib (INC280), a c-Met inhibitor, is investigated in a phase Ib/II clinical trials to assess its safety, dose and anti-tumor activity in patients with recurrent GBM with *PTEN* loss or *MET* alteration (NCT01870726). Another phase I study will evaluate the safety and dose of BKM120 in patients with newly diagnosed GBM when given in combination with radiotherapy and temozolomide (NCT01473901). Last but not least, a multi-center phase Ib/II study in patients with recurrent GBM is ongoing to determine the maximum tolerated dose (MTD) and therapeutic effect of BKM120 combined with carboplatin or lomustine (NCT01934361).

### XL147

Pilaralisib (XL147, SAR245408) is an orally bioavailable and reversible pan-class I_A_ PI3K inhibitor against p110α/δ/γ with IC_50_ of approximate 30 nM, but less potent upon p110β [43]. Apart from GBM, it exhibits dose-dependent anti-proliferative effects on breast cancer cells through inhibition on PI3K and Akt activation [44]. XL147 could induce an upregulation of HER3 expression and phosphorylation in HER2-overexpressing breast cancer cells, and it synergizes with trastuzumab or lapatinib to suppress xenograft growth [44, 45]. Currently, XL147 has entered phase I/II clinical trials in a variety of cancers including breast, endometrial, lymphoma and GBM [46–49]. A phase I exploratory pharmacodynamic study using XL147 and XL765 (Voxtalisib, a dual PI3K/mTOR inhibitor) was performed in patients with recurrent GBM prior to surgical resection (NCT01240460). Three cohorts of 21 patients were treated with different doses of XL147 and XL765 for >10 days, respectively. The mean tumor to plasma ratios of XL147 and XL765 were from 0.27 to 0.40, and treatment of these two drugs led to reduced S6 K1 phosphorylation and Ki67 expression, suggesting that they possess a moderate BBB-penetration capacity and a potential to inhibit GBM growth [49].

### PX-866

Sonolisib (PX-866) is an irreversible wortmannin analogue with a short half-life of 18.4 min in mouse, but showing a more persistent inhibitory effect on PI3K than wortmannin [50, 51]. It exhibits potent inhibition on A549 non-small cell lung cancer (NSCLC) cell xenograft growth, and overcomes the resistance of xenograft to EGFR inhibitor gefitinib [52]. Preclinical studies show that PX-866-induced toxicities include hyperglycemia and increased neutrophils, which could be reversed by the PPARγ agonist pioglitazone without affecting the anti-tumor activity of PX-866 [52, 53]. In 13 human cancer cell line-derived xenografts, tumors with *PTEN* loss or *PIK3CA* mutations are sensitive to PX-866, whereas those harboring RAS-activating mutation show resistance, suggesting that *RAS* mutation may be an important prognostic predictor of PX-866 for cancer treatment [54]. Recent studies using PX-866 alone or in combination with other drugs demonstrates pro-autophagic, anti-invasive and anti-angiogenic activities in GBM cell lines, as well as anti-tumor activities in intracranial GBM xenograft mice [55, 56]. PX-866 reduced GBM cell number in G1 phase via suppressing cyclin D1 expression and Rb1 activation, and induced autophagy through promoting the conversion of LC3-I to LC3-II. In addition, it induced a decrease of invasion rate and VEGF secretion in a panel of GBM cell lines. The growth of intracranial U-87 MG glioblastoma xenograft was inhibited leading to longer survival of mice [55]. Furthermore, combination of PX-866 and the dinuclear platinum compound BBR3610 displayed synergistic effects on reduction of GBM cell migration and extension of GBM xenograft mice survival [56]. As an orally bioavailable pan-PI3K inhibitor, PX-866 has entered into clinical trials for many cancers including ovarian, colorectal, prostate, head and neck (H&N) cancers, melanoma, NSCLC and GBM [57–62]. In a phase II study, 33 patients with recurrent GBM were orally administrated with PX-866. It was relatively well-tolerated but the overall RR was low. All participants had ceased therapy due to disease progression or toxicities including diarrhea, venous thromboembolism, fatigue, nausea, vomiting, and lymphopenia. 72.7% (24 of 33) of participants underwent disease progression, while 3.03% (1 of 33) of patients had partial response and 24.2% (8 of 33) of those achieved stable diseases. The median 6-month PFS was 17%. It is noteworthy that there was no significant association between clinical outcome and mutations of *PTEN*, *PIK3CA*, *PIK3R1*, or *EGFRvIII* [62].

### GDC-0941

Pictilisib (GDC-0941) is a derivative of the dual PI3K/mTOR inhibitor PI-103, but shows inhibition of class I PI3K with less potency to mTOR. It exhibits a comparable anti-proliferative activity to PI-103 against a panel of human cancer cell lines including U-87 MG GBM cells [63]. Furthermore, the oral bioavailability of GDC-0941 is up to 78% and it shows sustained and remarkable inhibition of Akt phosphorylation and tumor growth (98% inhibition) in subcutaneous U-87 MG xenograft mice [63]. Combination of GDC-0941 and a Bcl-2 family inhibitor ATB-263 displayed synergistic effects on loss of mitochondrial membrane potential, induction of GBM cell apoptosis and suppressing sphere formation in GBM stem-like cells, via decreasing Akt phosphorylation and Mcl-1 expression [64]. In addition, GDC-0941 also synergizes with a natural compound B10, a glycosylated derivative of betulinic acid, to inhibit GBM cell viability, enhance lysosomal compartments and induce lysosomal membrane permeabilization through increasing TFEB and LAMP1 expression [65]. GDC-0941 and another PI3K inhibitor GNE-317 were used in a preclinical study to investigate their BBB penetration properties and cerebral distribution in U-87 MG and GS2 intracranial GBM xenograft models. GNE-317 displayed superior BBB penetration in both U-87 MG and GS2 GBM models. In contrast, GDC-0941 barely penetrated through the intact BBB, leading to a higher level of GDC-0941 distribution in the core of U-87 MG tumor than in the healthy part of the brain [66]. This result suggests that it may be difficult for GDC-0941 to reach the distant part of GBM, thus being insufficient to treat GBM with intact BBB. An in-vivo study also shows that combination of GDC-0941, irinotecan, sunitinib and temozolomide doesn’t significantly prolong the survival of mice with GBM xenograft, possibly due to the poor BBB permeability of GDC-0941 [67]. Nevertheless, a phase IIb clinical trial in patients with recurrent GBM is ongoing to evaluate the anti-tumor activity of pembrolizumab (MK-3475, a PD-1 monoclonal antibody) alone or in combination of PI3K/Akt inhibitors including GDC-0941, NVP-BEZ235 and GDC-0068 (NCT02430363).

### Other novel pan-PI3K inhibitors

ZSTK474, AMG 511, GDC-0032 and BAY 80–6946 are more potent inhibitors against PI3K, and may be attractive options for GBM treatment. Both ZSTK474 and AMG 511 could suppress the proliferation of GBM cell lines and subcutaneous U-87 MG xenograft growth [68–70]. GDC-0032 exhibits potent anti-proliferative and anti-tumor activities in head and neck squamous cell carcinoma and uterine serous carcinomas cell lines and xenografts with *PIK3CA* mutations or amplifications [71, 72]. BAY 80–6946, a more potent inhibitor against p110α and p110δ, impedes cell proliferation and induces apoptosis in human breast, endometrial and hematologic cancer cell lines with *PIK3CA* mutations and/or HER2 overexpression [73]. Moreover, significant tumor regression was observed in animals bearing *HER2*-amplified and *PIK3CA*-mutated breast, colon or NSCLC tumors treated with BAY 80–6946 [73, 74]. Although there is no clinical trial of these inhibitors for GBM treatment yet, ZSTK474, GDC-0032 and BAY 80–6946 are currently in phase I-III trials in patients with advanced solid tumors and hematologic malignancies.

### Isoform-selective PI3K inhibitors

Selective inhibitors against p110 isoforms may display less off-target effects and toxicities, therefore, are alternative options for GBM treatment. In-vitro studies using traditional isoform-selective PI3K inhibitors show that class I_A_ PI3K isoforms play distinct roles in glioma progression. Inhibition of p110α by PIK-75 or A66 is sufficient to suppress GBM cell viability, migration and invasion, whereas inhibition of p110β by TGX-221 only blocks cell migration, and inhibition of p110δ by IC87114 or CAL-101 moderately impedes cell proliferation and migration [19, 23]. Taken together, due to the predominant role of p110α in RTK-mediated Akt signaling, inhibition of p110α may be an effective treatment for GBM.

Since *PIK3CA* mutations and *PTEN* loss/mutation are frequently found in GBM, isoform-selective PI3K inhibitors, especially against p110α and p110β, may have potentials for the treatment of GBM bearing these genetic mutations. Currently, several novel isoform-selective PI3K inhibitors including BYL719, MLN1117, CAL-101, GSK2636771 and CH5132799 have entered phase I/II clinical trials in patients with solid tumors and hematologic malignancies. However, no clinical trial of isoform-selective PI3K inhibitors has been carried out on GBM patients (Table 3).

Alpelisib (BYL719) is an orally available p110α inhibitor against with IC_50_ of 5 nM and a half-life of 2.9 h in rat [75]. It exhibits potent anti-proliferative and anti-tumor activities in a variety of cancers including breast cancer, nasopharyngeal carcinoma and lung squamous cell carcinoma (LSCC) in a *PIK3CA*-dependent manner [76–78]. It suppresses spheroid growth, invasion and epithelial-to-mesenchymal transition (EMT) in *PIK3CA*-mutated LSCC cells, and reduces tumor growth and mesenchymal phenotype in xenograft mice [78]. Cancer cells carrying *PIK3CA* mutations are more sensitive to BYL719, while tumors bearing *PIK3CA* amplifications or *PTEN* mutations only respond moderately. Thus, it suggests that *PIK3CA* alteration may be a positive prognostic predictor for the clinical use of p110α-selective inhibitors [79]. BYL719 is currently in phase I/II trials in patients with breast cancer, lung cancer, pancreatic cancer and squamous cell carcinoma etc. Another p110α-selective inhibitor MLN1117, and a p110α/γ inhibitor CH5132799 are also in phase I/II trials for advanced solid malignancies treatment [80, 81].

The selective p110β inhibitor GSK2636771 has entered into phase I/II trials for the treatment of advance solid tumors bearing *PTEN* loss or mutation (NCT01458067) [82]. Due to the high expression levels of p110δ and p110γ in leukocytes, the p110δ inhibitors CAL-101 and AMG319, as well as the p110δ/γ inhibitor INK1197 are currently in phase I/II clinical trials for patients with hematologic malignancies including leukemia, lymphoma and myeloma.

### Dual PI3K/mTOR inhibitors

Although mTOR plays important roles in regulating cancer cell growth, metabolism and protein synthesis, the allosteric mTOR inhibitor rapamycin (sirolimus) fails to be approved as an anti-cancer agent due to its immunosuppresive effects [83]. Subsequently, its structural analogues (rapalogs) such as RAD001 (everolimus) and CCI-779 (temsirolimus) with reduced immunosuppresive effects and improved pharmacological properties are designed and have been approved by FDA for the treatment of subependymal giant cell astrocytoma (SEGA) or renal cell carcinoma, suggesting a potential of mTOR inhibitors in cancer treatment [84, 85]. However, short-term treatment of rapamycin and its analogues inhibits mTORC1 activity and triggers a negative feedback loop to augment Akt activation (Fig. 1) [86, 87]. In addition, cancer cells still maintain mTOR activation even though the activities of PI3K and Akt are suppressed [88]. These crosstalk and feedback between mTOR and PI3K largely limit the therapeutic effects of mTOR or PI3K inhibitors. Therefore, dual PI3K/mTOR inhibitors such as NVP-BEZ235, XL765, GDC-0084 and PQR309 are produced and currently tested in clinical trials (Table 3).

### NVP-BEZ235

Dactolisib (NVP-BEZ235) is an orally bioavailable, reversible, ATP-competitive dual PI3K and mTORC1/2 inhibitor, which has been widely used in preclinical studies on various cancers including GBM, breast, colorectal and lung cancers [89–92]. It sensitizes GBM cells to temozolomide and radiotherapy in vitro and in vivo through reducing Akt activation, elevating pro-apoptotic molecules Bax and caspase-3 expression, and blocking radiation-induced DNA damage repair [89, 93]. However, it is important to note that its inhibitory effect on Akt activation is reversible, thus, enhanced radiosensitization is only observed when U-87 MG cells are simultaneously treated with NVP-BEZ235 and ionizing radiation [94]. Furthermore, NVP-BEZ235 alone or in combination of MEK1/2 inhibitors UO126 or SL327 promotes cell differentiation to neuronal and glial lineages and suppresses the tumorigenic potential of GBM stem-like cells (GSLCs) [95, 96]. These findings indicate that NVP-BEZ235 plus temozolomide, radiotherapy or other inhibitors may be possible strategies for GBM treatment. NVP-BEZ235 has already entered phase I/II clinical trials in a number of cancers including pancreatic neuroendocrine tumor, breast cancer, prostate cancer and leukemia [97–99]. In a phase I dose-escalation study of patients with advanced solid tumors, NVP-BEZ235 was generally well-tolerated with mild dose-limiting toxicities (DLTs) including mucositis, hyperglycemia, dehydration, fatigue and thrombocytopenia [98]. A phase Ib study in patients with advanced solid tumors including GBM showed that combination of NVP-BEZ235 and everolimus exhibited limited efficacy and intolerable toxicities including fatigue, diarrhea, nausea, mucositis, and elevated liver enzymes [99]. NVP-BEZ235 is also being applied in combination with MK-3475 in GBM patients in a phase IIB clinical trial mentioned above (NCT02430363).

### XL765

Voxtalisib (XL765, SAR245409) is also a potent ATP-competitive, orally bioavailable, BBB-permeable PI3K/mTOR inhibitor with high inhibition on p110γ and an additional inhibition against DNA protein kinase [100]. It exhibits robust anti-proliferative activity in a panel of GBM cell lines including U-87 MG, U-251 MG and U-373 MG via decreased phosphorylation of Akt, GSK3β and p70S6 K, reduced expression of cyclin D, and G1-cycle arrest. Distinct inhibitory effects of XL765 on cell proliferation are noted toward cancer cells bearing diverse genetic alterations. Cell lines harboring *PIK3CA* mutations or amplifications but without *RAS* mutations are most sensitive to XL765, whereas cells with *RAS* mutations are relative insensitive to XL765 even if they have *PIK3CA* mutations. Furthermore, XL765 alone or in combination of temozolomide markedly suppresses growth of subcutaneous or intracranial GBM xenograft, and prolongs survival of tumor-bearing nude mice, indicating its potent anti-GBM activity and BBB penetration capacity [100, 101]. In a phase I safety and dose-escalation study, drug-related toxicities of XL765 are tolerable in patients with solid tumors. They included nausea, diarrhea, vomiting, decreased appetite plus increased ALT/AST [102]. A phase I dose-escalation study in high-grade glioma patients using combination of XL765 with temozolomide, with or without concurrent radiotherapy, showed similar treatment-related adverse effects plus thrombocytopenia [103]. The BBB penetration capacity of XL765 was demonstrated in another phase I study in patients with recurrent GBM. S6 K1 phosphorylation and Ki67 expression were also reduced by XL765 (NCT01240460) [49].

### GDC-0084

GDC-0084 (RG7666) is a novel BBB-penetrating PI3K/mTOR inhibitor displaying a high brain-to-plasma ratio (1.9–3.3). It remarkably impedes the proliferation of five GBM cell lines and suppresses the growth of U-87 MG GBM xenografts through decreasing Akt phosphorylation [104]. In patients with progressive or recurrent high-grade glioma, the most frequent adverse events were fatigue, hyperglycemia, nausea, hypertriglyceridemia, rash, hypophosphatemia, decreased appetite, diarrhea, and stomatitis. High brain-to-plasma ratio and tumor-to-plasma ratio of GDC-0084 also suggested a uniform distribution throughout the brain (NCT01547546) [105].

### PQR309

PQR309 is a novel, ATP-competitive, BBB-penetrating pan-PI3K/mTOR inhibitor with potent inhibitory effects on Akt and ribosomal protein S6 phosphorylation. In a PC-3 prostate tumor xenograft model, PQR309 has been shown to be oral bioavailable, and it effectively inhibits PI3K/Akt signaling and reduces tumor growth [106]. Phase I studies of PQR309 in patients with advanced solid tumors show that and the most frequent adverse events are fatigue, hyperglycemia, nausea, diarrhea, constipation, rash, anorexia and vomiting. Concentration-dependent hyperglycemia has been confirmed as a mechanism-based toxicity of PQR309 (NCT01940133, NCT02483858) [107, 108]. A non-randomized phase II study in patients with progressive GBM is ongoing to evaluate the safety, efficacy, pharmacokinetics and pharmacodynamic effects of PQR309 (NCT02850744).

Other novel dual PI3K/mTOR inhibitors including NVP-BGT226, GSK2126458, GSK1059615, GDC-0980, VS-5584, PF-04691502 and PKI-587 have entered phase I/II clinical trials for advanced solid tumors. However, no clinical study on these inhibitors has been carried out in patients with GBM.

### Combination of PI3K and other molecules’ inhibitors

Considering that inhibition of PI3K isoforms might lead to compensatory activation of other signaling pathways, or the feedback, and subsequent compromise of the inhibitory effects, GBM patients may benefit from combination treatment strategies by dual inhibition of PI3K and other molecules.

Activation of RTKs not only drives PI3K/Akt signaling activation, but also stimulates other signaling pathways including MAPK, NF-κB and STAT3. It is noteworthy that, *PIK3CA* and *EGFRvIII* mutations often lead to PI3K activation independent of RTKs such as EGFR, HER2, PDGFR, VEGFR and c-Met, suggesting that inhibition of RTKs alone is not sufficient to obstruct PI3K/Akt signaling. Evidence shows that overall alteration (including amplification, mutation, rearrangement and altered splicing) of EGFR in TCGA GBM samples is up to 57.4% [4]. Therefore, PI3K inhibitors may need to work with the inhibitors or monoclonal antibodies of RTKs for cancer treatment. Preclinical studies show that combined inhibition of PI3K and EGFR displays synergism in breast cancer in vitro and in vivo, leading to retarded cell growth and tumor regression [109, 110]. Compared with single inhibitor alone, combination of a dual PI3K/mTOR inhibitor PF-05212384 and a pan-ERBB inhibitor PF-00299804 (dacomitinib) induces more apoptosis of GBM cells harboring both *EGFR* amplification and PI3K activation [111]. Currently, combination of PI3K inhibitors and RTKs inhibitors has become a therapeutic strategy for cancer treatment in clinical studies. A phase Ib study of BKM120 and trastuzumab (a HER2 antibody) in patients with *HER2* ^*+*^ or trastuzumab-resistant breast cancer demonstrates that this combination is generally well-tolerated and it inhibits activation of both PI3K/Akt and ERK pathways [112]. In a phase I dose-escalation study in patients with solid tumors, safety and efficacy of XL147 plus an EGFR monoclonal antibody erlotinib were evaluated. The combination exhibited limited antitumor activity and similar toxicities as with XL147 alone [113]. With respect to GBM patients, a phase Ib/II study of BKM120 plus a c-Met inhibitor INC280 in patients with recurrent GBM bearing *PTEN* loss or *MET* alteration is still ongoing (NCT01870726).

PI3K/Akt and MAPK pathways interact with each other to build a signaling network including convergence, crosstalk, and feedback loops. Ras is capable of interacting with the catalytic isoforms of PI3K to activate PI3K/Akt pathway, while it also serves as a small G-protein to activate MAPK pathway [114]. Therefore, combination therapies targeting PI3K/Akt and MAPK pathways may have synergism in cancers harboring *RAS* mutations. Evidence shows that mice with *K-RAS* mutated lung cancer well respond to the combination of NVP-BEZ235 and a MEK inhibitor ARRY-142886, rather than NVP-BEZ235 alone [115]. Moreover, combination of BKM120 and MEK inhibitors exhibits decreased glioblastoma cell viability and prolonged survival of mice with intracranial xenograft, through downregulating the phosphorylation of ERK, Akt and p70S6 K [116]. Combination of NVP-BEZ235 and MEK1/2 inhibitors UO126 or SL327 displays synergistic inhibitory effect on self-renewal and tumorigenic capacities of GBM stem-like cells (GSLCs), and prolongs the survival of mice with GSLC xenograft [96]. In addition, PI3K/Akt and MKK4/JNK pathways also co-operate to regulate cancer cell survival, migration and invasion [117]. Our previous study showed that concurrent inhibition of PI3Kβ and JNK synergistically suppresses GBM cell proliferation and migration, and tumor growth in U-87 MG xenograft mice [19]. A phase I trial shows that patients with advanced solid tumors receiving combination therapies targeting PI3K/Akt and Ras/Raf/MEK/ERK pathways have better prognosis in response to *PTEN* deletions and *KRAS*/*BRAF* mutations [118]. BKM120 in combination with the MEK1/2 inhibitor trametinib (GSK1120212) produced clinical benefits to patients with *KRAS*-mutant ovarian cancer [119].

Accumulating evidence supports that combined inhibition of PI3K and Hedgehog (Hh) pathways’ molecules such as Smoothened (Smo) is superior to single agent alone [120, 121]. Activation of PI3K/Akt signaling is accompanied by sonic hedgehog (Shh) signaling activation in PTEN-deficient GBM cells [120]. Growth factor-induced activation of Hh signaling positively correlates with Akt phosphorylation level in esophageal cancer and breast cancer, while inhibition of PI3K activity decreases Smo and Gli1 expression, suggesting that Hh signaling activation may be partially activated through PI3K/Akt signaling [122, 123]. In addition, loss of *PTEN* confers medulloblastoma cells resistance to Hh pathway inhibitor GDC-0449 [124]. These findings indicate that there is a crosstalk between PI3K/Akt and Hh pathways, and combined inhibition of these pathways may have synergistic outcomes. Evidence shows that combination of BKM120 and a Smo inhibitor LDE225 significantly reduces tumor growth and increases apoptosis in intracranial GBM xenografts [120]. This combination treatment in patients with advanced solid tumors including recurrent GBM is generally tolerable with mild adverse events such as increased ALT/AST, blood creatine phosphokinase and alkaline phosphatase, hyperglycemia, aphasia, nausea and fatigue (NCT01576666) [125]. In addition, combination of NVP-BEZ235 and LDE225 also shows synergistic inhibitory effects on self-renewal capacity of pancreatic cancer stem cells (CSCs) and their growth in nude mice [121].

### Clinical implications

To date, huge breakthroughs in understanding the central role of PI3K signaling in cancer have been achieved, suggesting an effective therapeutic approach for GBM via targeting PI3K. However, only a small number of PI3K inhibitors could step into clinical trials for GBM treatment, showing their limited effects on tumor regression at tolerated doses. Activation of compensatory pathways, drug toxicity and blood–brain barrier have posed challenges to further development of PI3K inhibitors in clinical trials. Firstly, blockade of a single pathway generally gives rise to the compensatory activation of other pathways to relieve the inhibitory effects and maintain tumor cell survival [126]. Although PI3K/Akt signaling plays an essential role in the survival and motility of tumor cells, GBM cells are not completely addictive to this signaling pathway. Moreover, another challenge is to translate the preclinical findings to clinical activity and improve the therapeutic effects of PI3K inhibitors at acceptable tolerability in GBM patients. Although pharmacological inhibition of PI3K suppresses GBM tumor growth and demonstrates favorable outcomes in in-vitro experimental and in-vivo preclinical studies, the clinical data show that their therapeutic effects on GBM patients do not reach the expectations, mostly with stable diseases [40, 42, 62, 103]. Indeed, high doses or long-period treatment of PI3K inhibitors may not be tolerated in GBM patients due to their on-target or off-target toxicities, leading to the low expectations at acceptable doses. The majority of PI3K inhibitors in the clinical studies exhibit adverse effects including fatigue, hyperglycemia, nausea, vomiting and increased ALT/AST [62, 102, 105]. In addition, the efficacy of PI3K inhibitors on GBM patients is largely limited by BBB, which is heterogeneously disrupted in GBM areas [127]. BBB integrity is completely compromised in the central regions of GBM, whereas it is slightly undermined in the peripheral areas with more invasive properties [128]. This intact BBB could prevent drugs delivering to invasive GBM cells, leading to cell survival and tumor recurrence. Therefore, rational combination with other molecule’s inhibitors or other therapeutic approaches, and discovery of more specific PI3K inhibitors or an effective drug-delivery system with BBB permeability are required to improve the therapeutic effects of PI3K inhibitors for GBM.

Current clinical trials on GBM patients tend to use pan-PI3K and dual PI3K/mTOR inhibitors rather than isoform-selective PI3K inhibitors. Although the latter shows less off-target effects and toxicities, their clinical efficacy and application are largely limited by various genetic alterations such as *PIK3CA, RAS* and *PTEN* mutations, seen in patients. In order to maximize the applications of these specific inhibitors, future therapeutic strategies should include the molecular pathological diagnosis of GBM patients like *PIK3CA*, *RAS* mutations and *PTEN* loss/mutation prior to drug administration.

## Conclusions

Targeting PI3K signaling as a therapeutic approach for cancer treatment has been discussed for more than a decade based on a solid foundation of experimental and preclinical studies. Recently, the p110δ inhibitor CAL-101 has been approved by FDA for certain types of lymphoma, offering hopes of PI3K inhibitors for cancer treatment [129]. Due to many challenges, clinical data do not favor PI3K inhibitors in GBM treatment, suggesting that targeting PI3K alone is not sufficient to treat GBM.

The future strategies to promote the potential of PI3K inhibitors for GBM treatment need to focus upon: [1] identifying genetic alternations such as *PI3KCA* mutations, *PTEN* mutations/loss and *RAS* mutations prior to the treatment regime; [2] rational combinations with other molecules’ inhibitors or other therapies, on the basis of understanding of the crosstalks between PI3K and other signaling molecules/pathways; [3] employing a BBB-permeable drug delivering system specifically targeting GBM cells to decrease toxicities on normal cells. Implement of these ideas may guide us in the right directions and develop more effective therapeutic approaches for GBM treatment.

## Abbreviations

4EBP: Eukaryotic initiation factor 4E–binding protein
ALT: Alanine aminotransferase
AST: Aspartate transaminase
BBB: Blood–brain barrier
CR: Complete remission
CSC: Cancer stem cell
DLT: Dose-limiting toxicity
EGFR: Epidermal growth factor receptor
EMT: Epithelial-to-mesenchymal transition
GBM: Glioblastoma multiforme
GPCR: G protein-coupled receptor
GSLC: Glioblastoma stem-like cell
H&N: Head and neck
HCC: Hepatocellular carcinoma
Hh: Hedgehog
IC_50_: Half maximal inhibitory concentration
LSCC: Lung squamous cell carcinoma
MTD: Maximum tolerated dose
mTOR: Mammalian target of rapamycin
mTORC: mTOR complex
NSCLC: Non-small cell lung cancer
OS: overall survival
PDK-1: Phosphoinositide-dependent kinase 1
PFS: Progression-free survival
PHLPP: PH domain and leucine rich repeat protein phosphatase
PI3K: Phosphatidylinositol 3-kinase
PIP2: Phosphatidylinositol 4,5-bisphosphate
PIP3: Phosphatidylinositol 3,4,5-triphosphate
PR: Partial remission
PTEN: Phosphatase and tensin homolog deleted on chromosome 10
RR: Response ratio
RTK: Receptor tyrosine kinase
Smo: Smoothened
TCGA: The Cancer Genome Atlas
TRAIL: Tumor necrosis factor-related apoptosis inducing ligand
TSC: Tuberous sclerosis complex
